# SLA-constrained service selection for minimizing costs of providing composite cloud services under stochastic runtime performance

**DOI:** 10.1186/s40064-016-1938-6

**Published:** 2016-03-08

**Authors:** Kuo-Chan Huang, Mu-Jung Tsai, Sin-Ji Lu, Chun-Hao Hung

**Affiliations:** Department of Computer Science, National Taichung University of Education, No. 140, Min-Shen Road, Taichung, Taiwan

**Keywords:** Composite cloud service, Service selection, Cost minimization, Stochastic performance, Service level agreement

## Abstract

Composite cloud services based on the methodologies of Software as a Service and Service-Oriented Architecture are transforming how people develop and use software. Cloud service providers are confronting the service selection problem when composing composite cloud services. This paper deals with an important type of service selection problem, minimizing the total cost of providing a composite cloud service with respect to the constraints of service level agreement (SLA). Two types of SLA are considered in the study: per-request-based SLA and ratio-based SLA. We present three service selection approaches for dynamic cloud environments where services’ performance might vary with time. The first two are iterative compound approaches for per-request-based SLA and the third approach is a one-step method for ratio-based SLA based on the Chebyshev’s theorem and nonlinear programming. Experimental results show that our approaches outperform the previous method significantly in terms of total cost reduction.

## Background

Cloud has become the most promising next-generation computing platform recently, and is usually divided into three layers of services (Buyya et al. 2008; Rai et al. 2015): Infrastructure as a Service (IaaS), Platform as a Service (PaaS), and Software as a Service (SaaS). The concept of SaaS (Jing and Zhang 2010) has caused a revolution in how we develop and use software applications. Service-Oriented Architecture (SOA) (Jing and Zhang 2010) is one of the important underlying technologies for developing SaaS applications, which are usually composite cloud services in the sense that they are developed by composing several existing services together. Software development methodologies based on the new models of SaaS and SOA are expected to bring a lot of benefits for both developers and users, such as fast and flexible development as well as greater market opportunities for software components.

However, before the benefits to be realized, developers of composite cloud services are confronting several new issues which were not seen in developing traditional stand-alone or simple web-based applications. The service selection problem (SSP) (Beran et al. 2012) is one of such important issues, dealing with how to effectively select appropriate ones among existing services for putting them together to develop new composite cloud services with some Quality of Service (QoS) goals in mind.

Usually, there will be service level agreements (SLA) between users and cloud service providers, which define the contracted performance of concerned QoS aspects and the penalties for violation of the contracted QoS. Therefore, for cloud service providers, an important kind of SSP is to minimize the cost with respect to the constraints of SLA, where the cost includes both usage costs of constituent services and penalties for SLA violation. In this paper, we focus on one important QoS aspect, the response time of the entire composite cloud service, and develop service selection approaches to minimizing the total costs for service providers under the regulation of SLA.

In most previous studies (Huang et al. 2009; Menasce et al. 2008), the SSP was addressed based on deterministic performance parameters. However, in practice we know that most performance parameters, e.g. service response time, are not always deterministic and rather follow a stochastic distribution in a dynamic cloud environment. To address the gap between most existing studies and the reality, in this paper, we propose three service selection approaches for dynamic cloud environments where services’ response time varies stochastically due to dynamically changing workload or other uncertainties. We consider two common types of SLA in the study: per-request-based SLA and ratio-based SLA.

The proposed approaches were evaluated with a series of simulation experiments and compared to the method in (Schuller et al. 2012), which tries to reduce SLA violation by choosing services with smaller standard deviation of response time in the adaptation step of the iterative service selection process. In our approaches, we take into consideration not only standard deviation of service response time but also services’ costs and mean response time when selecting services, in order to effectively reduce the total costs under the SLA constraints. The experimental results indicate that our approaches can outperform the previous method (Schuller et al. 2012) significantly in terms of total cost reduction in typical scenarios.

The remainder of this paper is organized as follows. “Related work” section discusses related works on the SSP. We present our three service selection approaches in “Service selection under stochastic performance” section. “Experiments and performance evaluation” section evaluates the proposed approaches and compares it with the method in (Schuller et al. 2012). “Conclusions” section concludes this paper.

## Related work

As cloud computing emerges, SaaS applications based on the SOA (Jing and Zhang 2010) methodology have become a promising direction for future development and usage of software and have received a lot of research attentions. The SaaS- and SOA-related research works in the literature span a variety of different issues, including execution platforms and runtime environments (Czarnul 2013), service discovery (Wang et al. 2015; Zisman et al. 2013), service ranking and recommendation (Rong et al. 2015), service selection (Trummer et al. 2014; Wang et al. 2014), service replacement (Hu et al. 2007), service allocation (Rebeish and Bahsoon 2013), service composition (Nagamouttou et al. 2015), service orchestration (Rosario et al. 2008), service choreography (Wang and Pazat 2013), service deployment (Mao et al. 2013), service security (Bernardo 2013), and other issues. In this paper, we focus on the issue of service selection which deals with the challenge regarding optimally selecting a service for each task in a composite cloud service from several functionally equivalent ones with an optimization objective for the entire composite cloud service under some QoS constraints.

Most previous studies on service selection assumed deterministic QoS parameters and proposed solutions based on different optimization techniques, such as genetic algorithms (Iordache and Moldoveanu 2013), ranking chaos optimization (Laillia et al. 2013), particle swarm optimization (Liaoa et al. 2014), Markov model (Singh and Pattanaik 2013), or heuristic approaches (Beran et al. 2012). However, in practice we know that most performance parameters, such as service response time, are not always deterministic, but rather follow a stochastic distribution in a dynamic cloud environment. Recently, there have been some research works starting to tackle the issues regarding stochastic QoS performance. The work in (Bruneo et al. 2013) aims to design a complete method to study the stochastic QoS of a composed web service at design time.

Liu et al. (2013) proposed an approach for web service dynamic composition based on global QoS constraints decomposition. The global QoS constraints are decomposed into local constraints by an algorithm named Culture Genetic Algorithm. Then, the best web services that can satisfy the local constraints are selected for each task during the running time. In contrast to the dynamic approach, our approaches in this paper complete the service selection process before the entire composite cloud service begin to serve users. The approach in Vineka et al. (2011) tries to maximize the quality of a single request and minimize the variance of the quality over time simultaneously, but doesn’t deal with the issues regarding costs and penalties of SLA violation.

Schuller et al. (2012) presented an integrated approach based on Integer Linear Programming (ILP) and simulation to address the SSP for complex workflows in conjunction with stochastic QoS parameters. The idea is to account for penalty cost, due to SLA violations, during the service selection process in order to reduce the impact of stochastic QoS behavior on total cost. The first two approaches of our work share the same goal, reduction of total costs of providing a composite cloud service, and a similar iterative service selection process with the work in (Schuller et al. 2012). However, our approaches consider both service usage costs and SLA violation penalties in the service adaptation step, and thus are more effective than the method in (Schuller et al. 2012) regarding total cost reduction.

## Service selection under stochastic performance

A solution to the SSP is a set of feasible assignments of specific services to the tasks within a composite cloud service, which can achieve the optimization goal and satisfy the QoS constraints.

### Composite cloud services

**Fig. 1:**
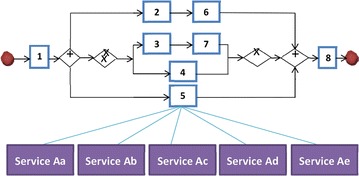
An example of composite cloud services

Based on the SOA development methodology, a composite cloud service is composed of a set of existing services, which might be developed by different providers and usually distributed over different (virtual) machines. A user’s request to a composite cloud service would start a distributed execution of its constituent services across several (virtual) machines according to the defined execution precedence among them. A composite cloud service can be viewed as a kind of workflow applications in the sense that the execution precedence among its constituent services can usually be described by a Directed Acyclic Graph (DAG), e.g. Fig. 1.

Figure 1 presents an example of composite cloud services in the commonly used BPMN notations (Sherry 2012) for illustrating the SSP, where ‘+’ represents an AND branch and ‘X’ indicates an XOR branch. In general, there are three types of execution dependencies among tasks within a composite cloud service: AND-block, XOR-block, and Sequence. An AND-block, between two ‘+’ symbols, consists of several paths where each of them must be executed and all the paths can be executed in parallel. An XOR-block, between two ‘X’ symbols, also contains a set of paths. However, each time only one of the paths will be executed, and which path to execute depends on the application status right before the XOR-block. The services on a path will be executed one-by-one serially, called the Sequence execution dependency.

The eight blue square blocks, each with an ID, in Fig. 1 represent the constituent tasks of the composite cloud service. Each task has to be bound to a specific service instance before the entire composite cloud service can be instantiated to provide services. For each task, there could be several functionally equivalent candidate services to choose. Providers of those services will offer their QoS and price attributes for composite cloud service developers to make their decisions on service selection. Table 1 shows an example of such QoS attributes and price information that will be considered in this paper. Since the response time of a service usually does not remain constant over time in a real dynamic cloud environment, the QoS attributes of each service contain both its average response time and standard deviation of response time.

**Table 1:** Services’ QoS and price attributes

ID	Average response time	Standard deviation	Price
*a*	110	1	500
*b*	120	2	400
*c*	130	3	300
*d*	140	4	200
*e*	150	5	100

On cloud platforms, users pay a fixed amount of money for using a specific cloud service according to the list price specified by the provider. There might be a SLA between users and the provider which specifies various QoS demands that the cloud service promises to satisfy. Once a cloud service violates the SLA requirement after its execution, there would be a penalty fee for the service provider. Since users pay a fixed amount of money for cloud services, the service provider would have a motivation to maximize his profits by reducing the costs as many as possible. The main costs of providing a composite cloud service come from two parts: the usage costs of the constituent services and the potential penalties for SLA violation. The penalty fee is assumed to be proportional to the exceeded service response time compared to that specified in SLA.

### Two iterative compound approaches for per-request based SLA

This section presents two iterative compound approaches to the SSP in a dynamic cloud environment adopting per-request-based SLA, where each user request is treated independently. Once a request’s response time exceeds the limit defined in the SLA, a corresponding penalty fee occurs for the service provider.

To cope with services’ stochastic performance, our approaches are based on an iterative process, similar to the structure in (Schuller et al. 2012), consisting of a deterministic optimization step using ILP (Conforti and Cornuejols 2014), a simulation step for evaluating potential SLA violation, and an adaptation step for service reselection. The three steps are repeated until a good enough solution is found based on some criteria.

At the first step, costs and average response time of services are used to formulate an optimization problem and then find a solution using ILP (Conforti and Cornuejols 2014). To prepare the formulation of an ILP optimization problem, we need to derive the expected QoS values of an entire composite cloud service from the attribute values of each constituent service. According to (Schuller et al. 2012), the following symbols are defined. The set of all tasks within a composite cloud service is labeled with *I*. The set of services functionally appropriate to execute a certain task *i* is labeled with *J*_*i*_. The decision variables *x*_*ij*_ ∈ {0, 1} indicate whether a service *j* is selected to conduct task *i*. Cost is represented by *c* and response time is indicated by *t*. Regarding branches in AND-block and XOR-block, we label the set of paths with *L*. Referring to the composite cloud service in Fig. 1, there are two paths within the XOR-block, and thus *L*_*xor*_ = {*p1*, *p2*}, where *p1* represents the path containing tasks 3 and 7, and *p2* for the path of task 4. The tasks within a branching are covered by the set *I*_*L*_ ⊆ *I*, whereas *I*_*l*_ ⊆ *I*_*L*_ represents the set of tasks within path *l*. *Sequence* is used to label the set of tasks which are not located within any branching structure. Based on the above notations, minimizing the cost of using constituent services can be represented by an optimization problem as the following formula.

$${ \hbox{min} }\left( {\mathop \sum \limits_{{i \in I_{s} }} \mathop \sum \limits_{{j \in J_{s} }} {\text{c}}_{ij} x_{ij} + \mathop \sum \limits_{{l \in L_{AND} }} \mathop \sum \limits_{{i \in I_{l} }} \mathop \sum \limits_{{j \in J_{si} }} c_{ij} x_{ij} + \mathop {\hbox{max} }\limits_{{l \in L_{XOR} }} \mathop \sum \limits_{{i \in I_{l} }} \mathop \sum \limits_{{j \in J_{si} }} c_{ij} x_{ij} } \right)$$

Table 2 is an example ILP formulation for the above optimization problem based on the worst-case aggregation functions (Schuller et al. 2012) in Table 3, taking the composite cloud service in Fig. 1 as an example. To make the formulation amenable to ILP, we introduce three new variables, i.e. *time1*, *time2*, and *cost*, for avoiding the max operators in the above formula and the worst-case aggregation functions, as shown in Table 2.

**Table 2:** Formulation of ILP

Goal	$\hbox{min} \left( {\sum\nolimits_{{i \in I_{s} }} {\sum\nolimits_{{j \in J_{s} }} {{\text{c}}_{ij} x_{ij} } + \sum\nolimits_{{l \in L_{AND} }} {\sum\nolimits_{{i \in I_{l} }} {\sum\nolimits_{{j \in J_{si} }} {c_{ij} x_{ij} + cost} } } } } \right)$
Conditions	$\sum\nolimits_{{i \in I_{s} }} {\sum\nolimits_{{j \in J_{s} }} {t_{ij} x_{ij} + tim{\text{e}}1 + {\text{time}}2 \le {\text{SLA}}} }$
	$$\forall {\text{l}} \in L_{AND} \sum\nolimits_{{i \in I_{l} }} {\sum\nolimits_{{j \in J_{si} }} {t_{ij} x_{ij} \le time1} }$$
	$$\forall {\text{l}} \in L_{XOR} \sum\nolimits_{{i \in I_{l} }} {\sum\nolimits_{{j \in J_{si} }} {t_{ij} x_{ij} \le time2} }$$
	$$\forall {\text{l}} \in L_{XOR} \sum\nolimits_{{i \in I_{l} }} {\sum\nolimits_{{j \in J_{si} }} {C_{ij} x_{ij} \le cost} }$$
	$$\sum\nolimits_{{j \in J_{i} }} {x_{ij} = 1}$$

**Table 3:** Worst-case aggregation functions for response time and costs

	Sequence	AND-block	XOR-block
Response time	$\sum\nolimits_{{i \in I_{s} }} {\sum\nolimits_{{j \in J_{s} }} {t_{ij} x_{ij} } }$	$\mathop {\hbox{max} }\limits_{{l \in L_{AND} }} \sum\nolimits_{{i \in I_{l} }} {\sum\nolimits_{{j \in J_{si} }} {t_{ij} x_{ij} } }$	$\mathop {\hbox{max} }\limits_{{l \in L_{XOR} }} \mathop \sum \limits_{{i \in I_{l} }} \mathop \sum \limits_{{j \in J_{si} }} t_{ij} x_{ij}$
Cost	$\sum\nolimits_{{i \in I_{s} }} {\sum\nolimits_{{j \in J_{s} }} {c_{ij} x_{ij} } }$	$\sum\nolimits_{{l \in L_{AND} }} {\sum\nolimits_{{i \in I_{l} }} {\sum\nolimits_{{j \in J_{si} }} {c_{ij} x_{ij} } } }$	$\mathop {\hbox{max} }\limits_{{l \in L_{XOR} }} \sum\nolimits_{{i \in I_{l} }} {\sum\nolimits_{{j \in J_{si} }} {c_{ij} x_{ij} } }$

The first step described in the above simply concerns the cost of using constituent services, and doesn’t deal with potential penalty for SLA violation. The second step in the iterative process simulates the execution of an entire composite cloud service in a dynamic environment where the response time of each constituent service varies stochastically, using the services selected in the first step. To estimate the effects of services’ stochastic performance on penalty costs, the simulation will be conducted for many times and each constituent service’s response time might vary at different runs. The stochastic variation of each constituent service’s response time is determined by one of its QoS attributes: the standard deviation in Table 1. Therefore, in some runs the resultant response time of the entire composite cloud service might exceed the requirement of SLA, incurring penalty fees. The average cost of an entire composite cloud service across all the simulation runs will then be used to evaluate the appropriateness of the selected services.

Based on the simulation results of the second step, the third step makes an adaptation to the previous service selection. In (Schuller et al. 2012), for each iteration only one task within the composite cloud service is chosen for service reselection at the third step. This task is called *critical task* and is determined by the following Algorithm 1. The algorithm consists of a for-loop checking each task in the composite cloud service. For each task, the potential *benefit* of changing the selected service is determined by calculating the difference between the selected service’s standard deviation of response time, *σ*_*s*_, and the smallest standard deviation among other candidate services, as shown in line 3. A *weight, ω,* is assigned to each task, calculated by dividing the number of the simulation runs where the task was executed and the SLA was violated by the number of total runs. The calculation of *w* is represented by the computeWeight(*i*) function shown in line 4. The task with the highest weighted benefit is chosen as the critical task for the adaptation step, as shown in lines 5–8.

With the critical task determined, the adaptation step in (Schuller et al. 2012), as shown in the following Algorithm 2, tends to reduce SLA violation by excluding services with standard deviation larger than currently selected one from next iteration’s ILP optimization, as shown in lines 4 and 5 where the setBanned(*j*) function will exclude service *j* from next iteration’s service selection. We call this method Deviation-Driven Iterative Compound Approach (DD-ICA) hereafter in this paper.

However, choosing services with smaller standard deviation of response time does not always lead to reduced costs since those services might have higher prices. In the following, we propose two new adaptation methods for the iterative service selection process.

**Fig. 2:**
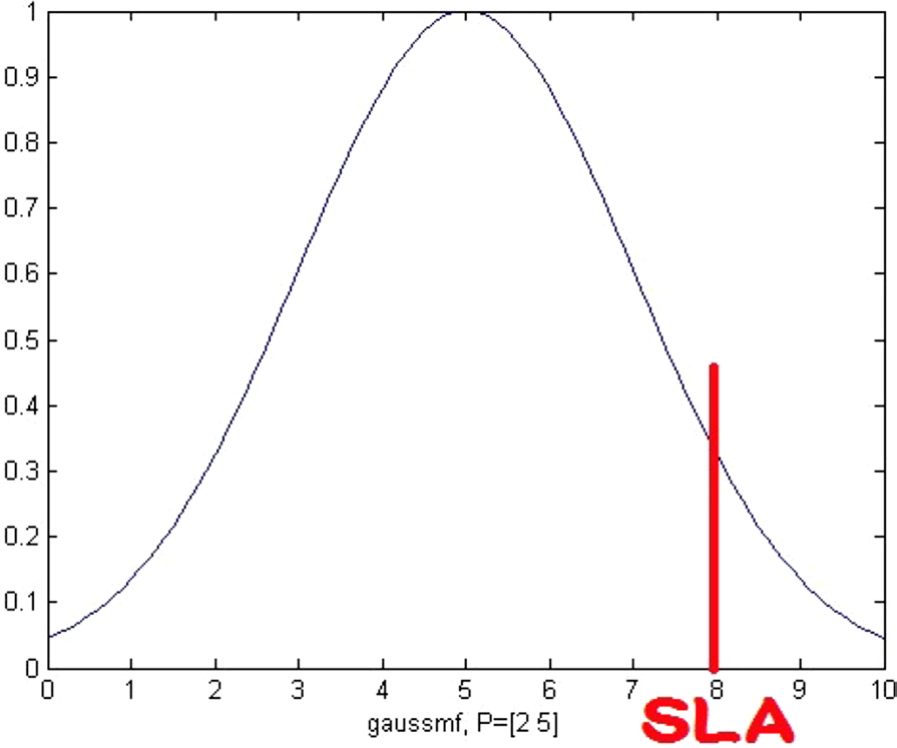
Normal distribution of service response time

The first method, as shown in Algorithm 3, is called Cost-Driven Iterative Compound Approach based on Chebyshev’s theorem (CD-ICA-Chebyshev), which tries to estimate the potential SLA violation cost based on Chebyshev’s theorem (Walpole and Myers 2012). Without loss of generality, we assume all services’ response time follows the normal distribution, as shown in Fig. 2, each with different mean values and standard deviation. According to the fundamental theorems of statistics, means and variances of linear combinations of random variables can be calculated (Walpole and Myers 2012). Therefore, after selecting a specific set of services, the mean and standard deviation of the response time of the entire composite cloud service can be calculated based on the QoS attributes of the constituent services. Then, we can estimate the probability that the response time of the composite cloud service exceeds what specified in SLA, i.e. the area to the right of the red line in Fig. 2, as follows.

Let *μ* + *kσ* = SLA, where *μ* and *σ* are the mean and standard deviation of the response time of the entire composite cloud service and SLA stands for the bound on response time in the SLA, the probability of SLA violation should be no more than 1/2*k*^2^ according to Chebyshev’s theorem (Walpole and Myers 2012). In contrast to DD-ICA (Schuller et al. 2012), our CD-ICA-Chebyshev approach considers both constituent services’ usage costs and the potential penalty incurred by response time deviation at the adaptation step, as shown in Algorithm 3, in order to minimize the total costs of running a composite cloud service.

In Algorithm 3, lines 1 and 2 calculate the mean response time and standard deviation of the entire composite cloud service based on the attributes of the constituent services. Line 3 computes the parameter *k* in Chebyshev’s theorem. Line 4 calculates the total cost of using the selected services. Lines 5–16 are similar to the adaptation step in DD-ICA (Schuller et al. 2012), as shown in Algorithm 2, but different in how to determine which services should be banned in the next iteration of service selection. The difference lies in lines 9–13 where for each candidate service of the critical task, the resultant performance of the entire composite cloud service, once using it, is calculated, and both service usage cost and potential SLA violation penalty are used to determine which candidate services to ban.

Our second approach is called Cost-Driven Iterative Compound Approach based on Integration (CD-ICA-Integration). The basic framework of this approach is similar to CD-ICA-Chebyshev. However, this approach tries to estimate the potential SLA violation cost in a more accurate manner than CD-ICA-Chebyshev. As shown in Algorithm 4, compared to CD-ICA-Chebyshev, the difference lies in lines 3–11, where the SLA violation cost, i.e. *p*, is computed by integrating the potential penalty across all possible response time which exceeds the requirement in SLA. In Algorithm 4, the response time, without loss of generality, is assumed to follow a normal distribution, i.e. $\frac{1}{{\sigma \sqrt {2\pi } }}e^{{ - \frac{{\left( {x - \mu } \right)^{2} }}{{2\rho^{2} }}}}$. However, this approach can be applied with other kinds of probability distributions, too.

The proposed service selection approaches conduct iterative execution of the above-mentioned three steps, i.e. ILP optimization, simulation of stochastic performance, and adaptation, controlled by two parameters, *greed* and *anneal*, as in (Schuller et al. 2012). The parameter *greed* determines how often the three steps are repeated as long as new iterations reduce total costs. Another parameter *anneal* controls for allowing worse solutions temporarily as starting points for further iterations.

### One-step nonlinear programming approach for ratio-based SLA

The two approaches presented in the previous section and the DD-ICA approach in (Schuller et al. 2012) are appropriate for per-request-based SLA, where SLA is applied to each single request and a penalty cost is incurred once the response time of a request exceeds the requirement in SLA. However, there is another kind of SLA, called ratio-based SLA in this paper, which is also commonly used in practice, e.g. Amazon EC2 (2015, https://aws.amazon.com/tw/ec2/sla/), where SLA is applied to a set of requests spanning a specific time period, e.g. a week or a month, and an SLA violation occurs when a predefined ratio of the requests cannot achieve the commitment defined in SLA. In this section, we propose a service selection approach suitable for ratio-based SLA, where service providers try to minimize their usage costs of constituent services provided that the SLA is not violated.

Compared to the iterative process in the approaches in “Two iterative compound approaches for per-request based SLA” section, this approach is a one-step method based on Chebyshev’s theorem (Walpole and Myers 2012) and nonlinear programming (Bertsekas 1999). The one-step approach takes into consideration the stochastic performance in the objective function of a nonlinear programming formulation. Based on statistics methods, the one-step approach doesn’t need to adopt a simulation procedure to estimate the effects of services’ stochastic runtime performance followed by an adaptation step to adjust service selection.

The nonlinear programming approach requires higher computational complexity than ILP. However, the advantage of this approach is its capability of enforcing an upper bound on the ratio of response-time-limit violation while minimizing the service usage costs. Many modern mathematical tools, such as MATLAB (Gilat 2014), can handle such kind of nonlinear optimization problems. In the experiments presented in “Experiments and performance evaluation” section, we implemented this service selection approach based on MATLAB’s related APIs. Assuming that the SLA requires no more than *r* % requests having their response time exceeding the bound defined in SLA, Table 4 shows the entire nonlinear programming formulation of the SSP.

**Table 4:** Formulation of nonlinear programming

Goal	${ \hbox{min} }\left( {\sum\nolimits_{{i \in I_{s} }} {\sum\nolimits_{{j \in J_{s} }} {{\text{c}}_{ij} x_{ij} } } + \sum\nolimits_{{l \in L_{AND} }} {\sum\nolimits_{{i \in I_{l} }} {\sum\nolimits_{{j \in J_{si} }} {c_{ij} x_{ij} } } } + \mathop {\hbox{max} }\limits_{{l \in L_{XOR} }} \sum\nolimits_{{i \in I_{l} }} {\sum\nolimits_{{j \in J_{si} }} {c_{ij} x_{ij} } } } \right)$
Conditions	*μ* = *mean response time of the entire composite cloud service calculated based on t*_*i,j*_ *and x*_*i,j*_
	*σ* = *standard deviation of response time of the entire composite cloud service calculated based on ti,j and xi,j*
	*bound* = *the limit on response time specified in SLA*
	$$k = \frac{bound - \mu }{\sigma }$$
	$$\frac{1}{{2k^{2} }} \le r\%$$
	$$\sum\nolimits_{{j \in J_{i} }} {x_{ij} = 1}$$

## Experiments and performance evaluation

This section presents a series of simulation experiments for evaluating the service selection approaches proposed in the previous section. The simulation experiments were conducted on our composite-cloud-service execution simulator developed in Java and using lp_solve 5.5.2.0 for linear programming (2015, http://lpsolve.sourceforge.net/5.5/) and MATLAB APIs for nonlinear programming (Gilat 2014).

### Experimental setup

Since it is not possible to test all probable configurations of composite cloud services, we choose to experiment with a representative one. Although there might be various configurations for composite cloud services, they are all composed of a limited set of primitive structures, e.g. the AND-structure, XOR-structure, and Sequence-structure in BPMN (Sherry 2012). Therefore, in the experiments, we used the composite cloud service configuration shown in Fig. 1 as input, which could be representative in the sense that it contains all the three primitive structures. We believe that the experimental results based on the configuration in Fig. 1 would be valid for most composite cloud service configurations although not all. Moreover, the configuration in Fig. 1 is also the composite cloud service configuration used for evaluating DD-ICA in (Schuller et al. 2012). Since we want to compare our approaches to DD-ICA (Schuller et al. 2012) in the experiments, we choose to use the same composite cloud service configuration as input.

In the experiments, each task of the composite cloud service has five candidate services to choose. We considered two common scenarios, shown in Table 5, regarding the relationship among the services’ three attributes: mean response time, standard deviation of response time, and price. In the first scenario, services’ average response time and standard deviation of response time are inversely proportional to their prices. This is a common scenario and reasonable pricing strategy in practice. In the second scenario, services’ average response time is inversely proportional to their prices, but the values of standard deviation of response time are distributed randomly due to dynamic, unexpected workload fluctuation. Each experiment conducted 1000 simulation runs to simulate the processing of 1000 requests under stochastic performance of constituent services. Among the 1000 requests simulated, only the results of the last 950 requests were used to compute the average cost in order to prevent the potential initialization bias problem. In addition, each experiment was repeated 15 times with different random number sequences and the average value was used for the experimental result in order to overcome the potential autocorrelation problem.

**Table 5:** Two common relationships among services’ QoS parameters and prices

	Average response time	Standard deviation	Price
Scenario 1	↑	↑	↓
Scenario 2	↑	Random	↓

### Performance results

**Fig. 3:**
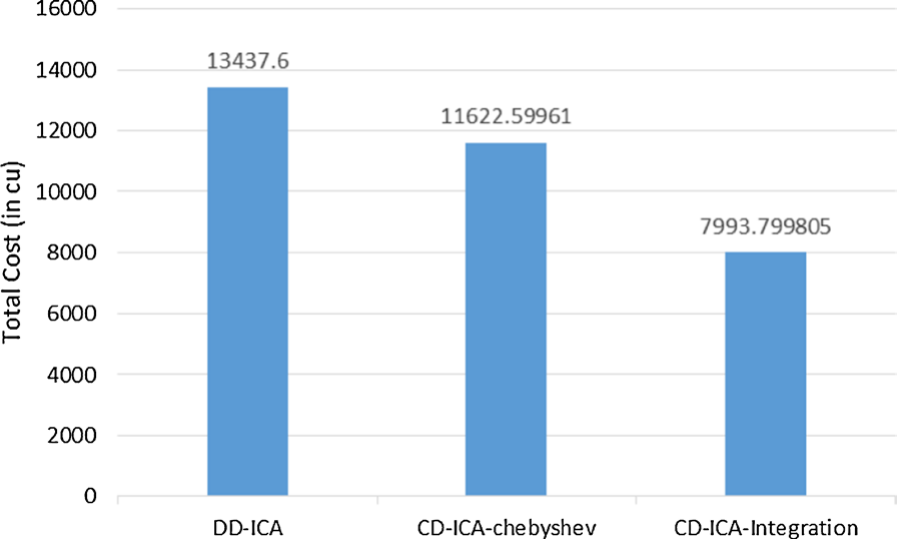
Total costs for scenario 1

Figure 3 compares our two service selection approaches for per-request-based SLA, i.e. CD-ICA-Chebyshev and CD-ICA-Integration, with DD-ICA (Schuller et al. 2012) for scenario 1 in terms of the total cost for providing the composite cloud service, including both constituent services’ usage costs and penalties for SLA violation, where cu represents the cost unit assumed in this study. The experimental results indicate that our two approaches outperform DD-ICA (Schuller et al. 2012) significantly, and CD-ICA-Integration performs the best among all approaches.

**Fig. 4:**
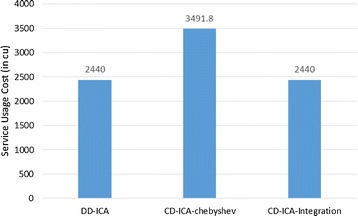
Service usage costs for scenario 1

**Fig. 5:**
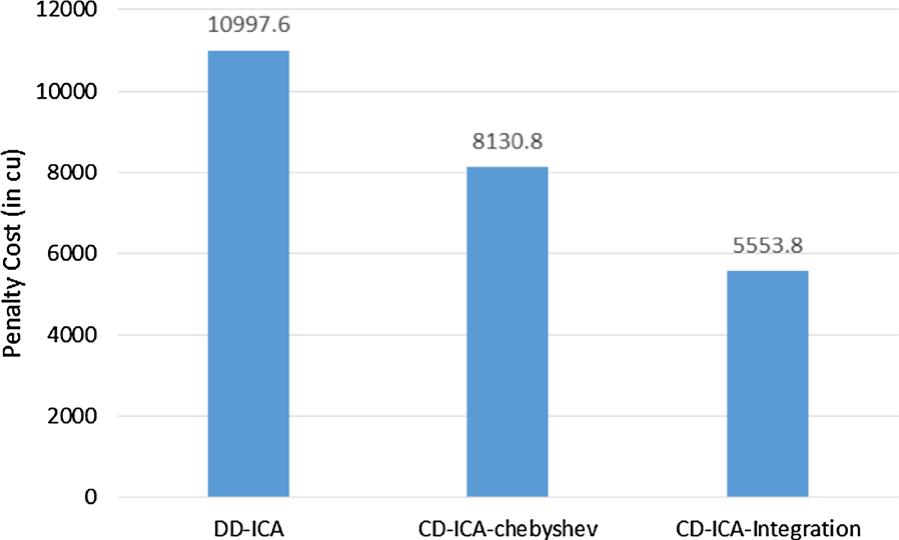
Penalty costs for scenario 1

Figures 4 and 5 elaborate on why CD-ICA-Integration achieves the smallest total cost by comparing the three approaches in terms of constituent services’ usage cost and penalty cost, respectively. The experimental results in the figures indicate that CD-ICA-Integration leads to a smaller penalty cost by selecting constituent services of higher prices, which have lower mean response time and standard deviation according to the row of scenario 1 in Table 5. CD-ICA-Integration has the capability of making better reconciliation between constituent services’ usage cost and penalty cost, and thus outperforms the other two approaches.

**Fig. 6:**
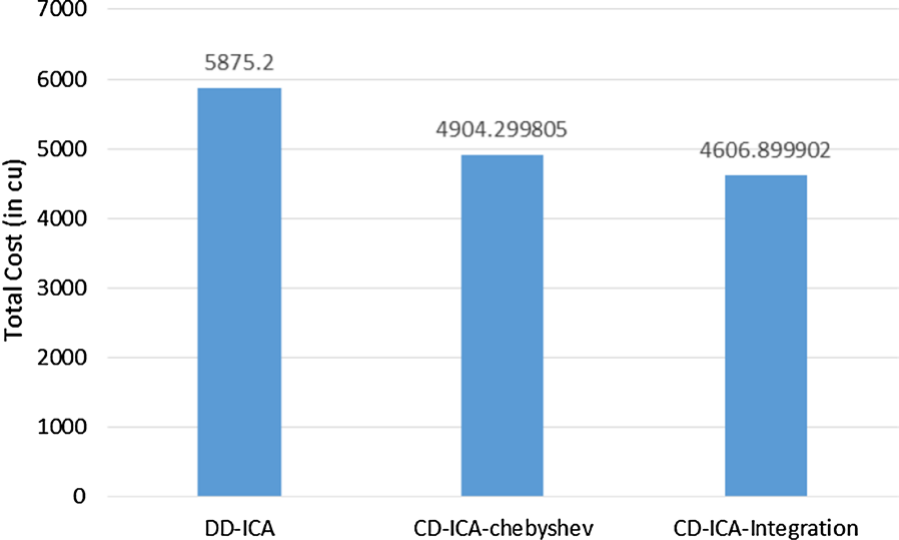
Total costs for scenario 2

Figure 6 presents the total cost comparison for scenario 2. Our approaches also outperform DD-ICA (Schuller et al. 2012) in this scenario, and CD-ICA-Integration still performs the best. However, the amount of achieved cost reduction in this scenario is less than that in scenario 1 due to the random correlation between standard deviation of service response time and service price.

**Fig. 7:**
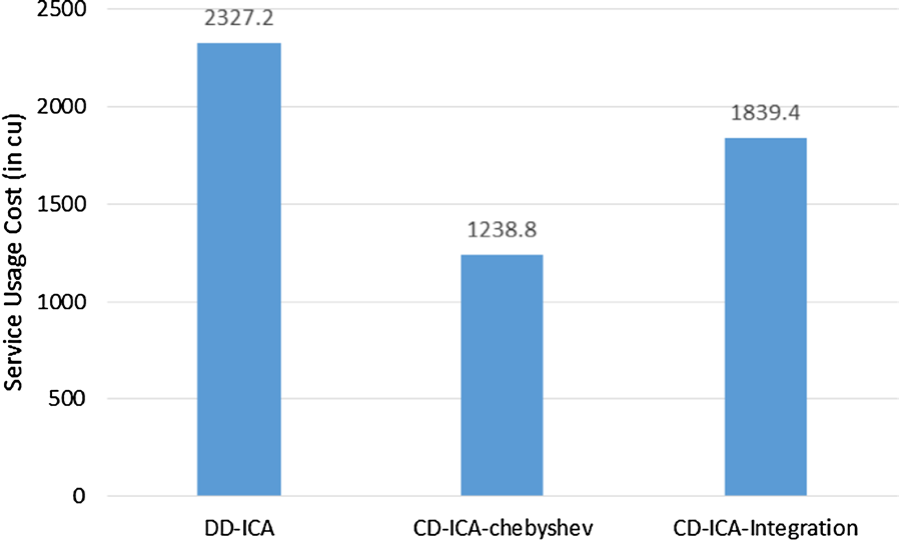
Service usage costs for scenario 2

**Fig. 8:**
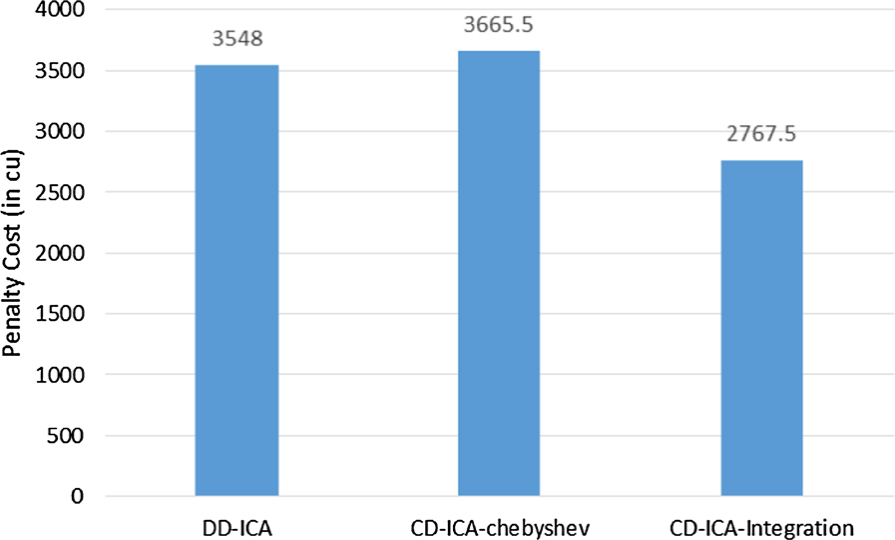
Penalty costs for scenario 2

Figures 7 and 8 compare the three approaches in terms of constituent services’ usage cost and penalty cost, respectively. In contrast to the experimental results for scenario 1, CD-ICA-Integration has the potential to achieve lower costs for both constituent services’ usage and penalty, compared to DD-ICA (Schuller et al. 2012), in scenario 2 as shown in Figs. 7 and 8, due to the random correlation between standard deviation of service response time and service price. For CD-ICA-Chebyshev, although it achieves the lowest service usage cost, as shown in Fig. 7, on the contrary, it leads to the highest penalty cost, resulting in a higher total cost than CD-ICA-Integration. The experimental results again demonstrate that CD-ICA-Integration has better capability of making reconciliation between constituent services’ usage cost and penalty cost.

**Fig. 9:**
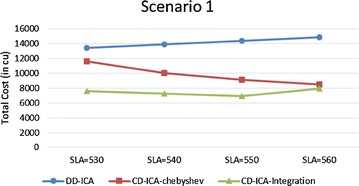
Total costs with different SLA values for scenario 1

**Fig. 10:**
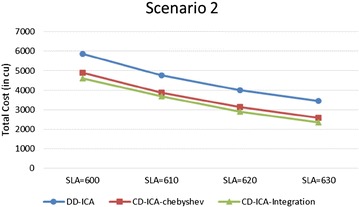
Total costs with different SLA values for scenario 2

In order to make thorough evaluation of the proposed approaches, the following presents a series of experimental results regarding different potential influence factors. Figures 9 and 10 compare the three service selection approaches across different SLA constraints on service response time in terms of total costs for providing the composite cloud service under the two scenarios, respectively. The experimental results indicate that our two approaches, CD-ICA-Chebyshev and CD-ICA-Integration, outperform the previous approach, DD-ICA (Schuller et al. 2012), significantly across different SLA values in both of the two scenarios. In scenario 1, the total cost difference between CD-ICA-Chebyshev and CD-ICA-Integration decreases as the SLA value increases, while in scenario 2, the difference between the three approaches remains nearly constant across different SLA values.

**Fig. 11:**
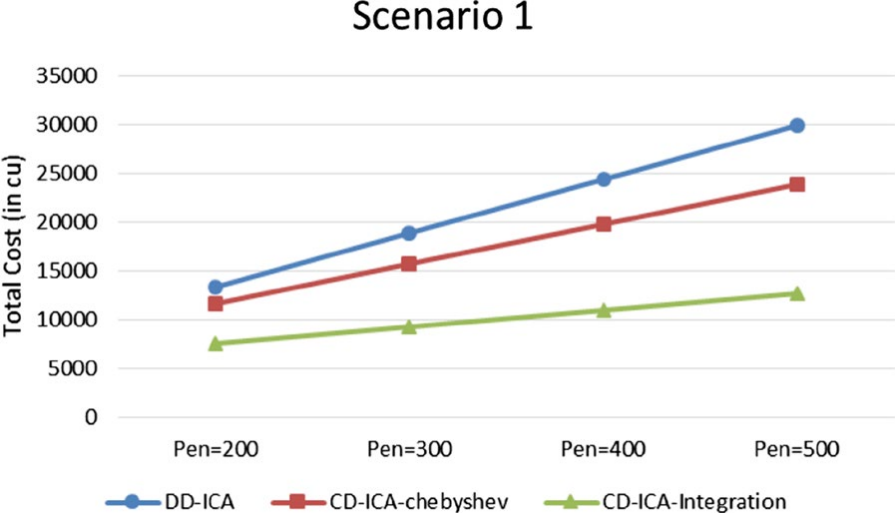
Total costs with different unit penalty cost for scenario 1

**Fig. 12:**
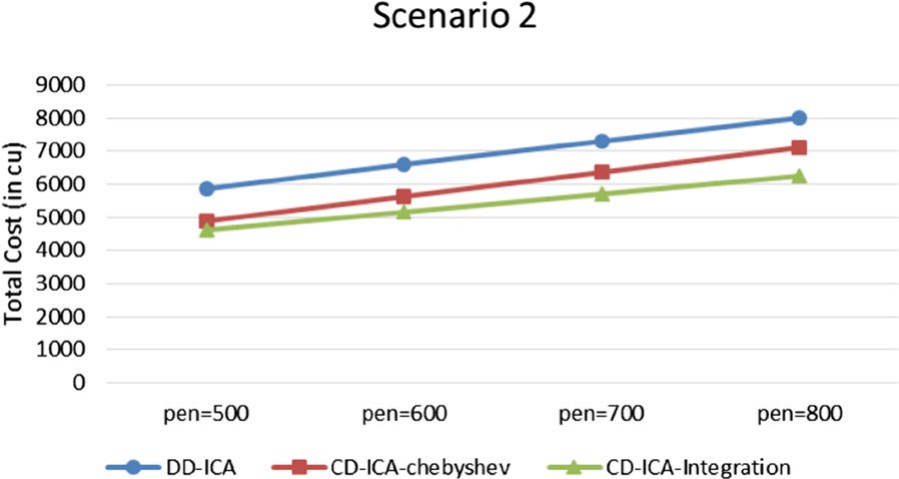
Total costs with different unit penalty cost for scenario 2

Figures 11 and 12 compare the three service selection approaches across different unit penalty costs, i.e. penalty per time unit, in terms of total costs under the two scenarios, respectively. The experimental results show that our two approaches outperform DD-ICA (Schuller et al. 2012) consistently across different unit penalty costs in both scenarios. The total cost difference between different approaches increases as the unit penalty cost grows. The increasing trend is particularly obvious for scenario 1 since where services’ average response time and standard deviation of response time have a clear inversely proportional relationship with their prices.

**Fig. 13:**
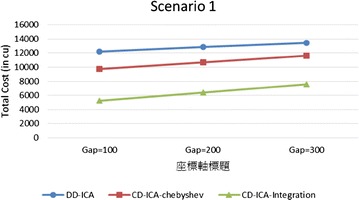
Total costs with different price gaps for scenario 1

**Fig. 14:**
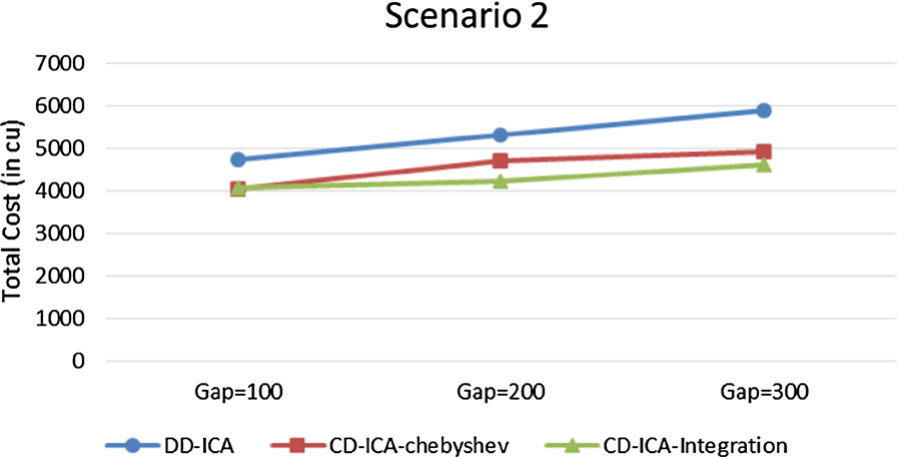
Total costs with different price gaps for scenario 2

Figures 13 and 14 compare the three service selection approaches across three situations which differ in the average price difference between services. The experimental results show that our approaches perform better than DD-ICA (Schuller et al. 2012) under all situations.

**Fig. 15:**
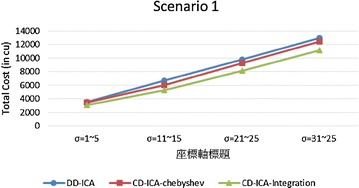
Total costs with different standard deviation of service response time for scenario 1

**Fig. 16:**
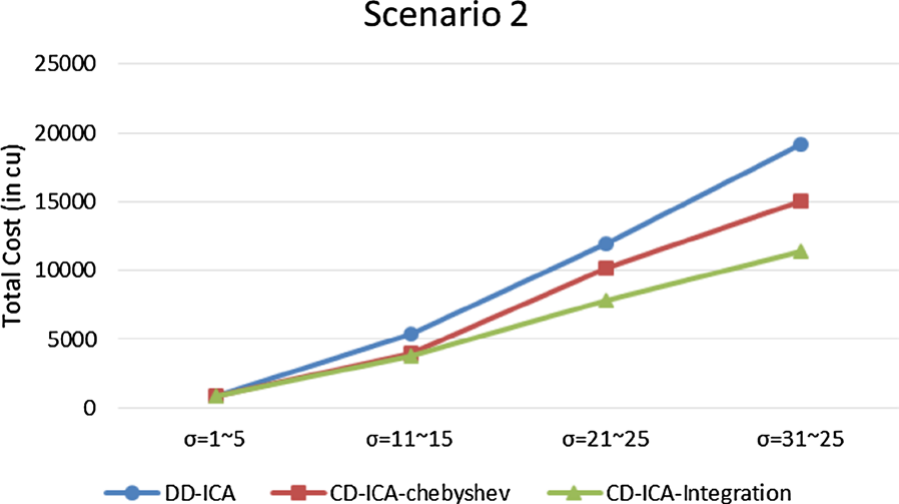
Total costs with different standard deviation of service response time for scenario 2

Figures 15 and 16 compare the three service selection approaches across different situations where constituent services have different ranges of standard deviation of service response time. The experimental results show that our approaches consistently outperform DD-ICA (Schuller et al. 2012) in all situations. The total cost reduction achieved by our approaches increases as the standard deviation of service response time grows. This increasing trend is particularly significant for scenario 2, where the relationship between the standard deviation of a service’s response time and its price is random, demonstrating CD-ICA-Integration has better capability of handling such complicated relationship among services’ different attributes.

Table 6 presents the experimental results of evaluating the capability of the One-Step Ratio-Based Nonlinear Optimization (OSRBNP) approach for limiting the ratio of violating the constraint on service response time specified in SLA. In the experiments, the allowable violation ratio is set to 5 % in SLA, representing that up to 50 violations are allowed among 1000 service requests. The experimental results in Table 6 demonstrate the advantage of OSRBNP over DD-ICA (Schuller et al. 2012), where OSRBNP leads to less than 50 violations as required by SLA, while DD-ICA (Schuller et al. 2012) cannot conform to the requirement of SLA.

**Table 6:** Number of service response time violation among 1000 requests

	DD-ICA	OSRBNP
Scenario 1	215	0
Scenario 2	235	2

## Conclusions

In this paper, we investigate the SSP in a dynamic cloud environment with stochastic performance variation. The goal is to provide composite cloud services at the minimal costs under SLA constraints. We propose three approaches in this paper. The first two are iterative compound approaches consisting of three steps: ILP optimization, simulation of stochastic performance, and adaptation. The third approach is a one-step method based on the Chebyshev’s theorem and nonlinear programming. The first two approaches, CD-ICA-Chebyshev and CD-ICA-Integration, were developed for per-request-based SLA, aiming for minimizing total cost by considering both constituent services’ usage costs and the potential penalty incurred by response time deviation at the adaptation step. The third approach, OSRBNP, was for ratio-based SLA, being able to enforce an upper bound on the ratio of response-time-limit violation while minimizing the constituent services’ usage cost.

The proposed approaches were evaluated with a series of simulation experiments and compared to the previous method, DD-ICA (Schuller et al. 2012), under two common scenarios which differ in the relationship among services’ QoS attributes and their prices. The experimental results indicate that our approaches outperform the previous method across different scenarios, achieving significant total cost reduction.
